# Bestrophin 3 Ameliorates TNFα-Induced Inflammation by Inhibiting NF-κB Activation in Endothelial Cells

**DOI:** 10.1371/journal.pone.0111093

**Published:** 2014-10-20

**Authors:** Wei Song, Zhen Yang, Ben He

**Affiliations:** 1 Department of Cardiology, Renji Hospital, School of Medicine, Shanghai Jiaotong University, Shanghai, China; 2 Department of Hypertension and Vascular Disease, the First Affiliated Hospital of Sun Yat-Sen University, Guangzhou, China; Ottawa Hospital Research Institute, Canada

## Abstract

Increasing evidences have suggested vascular endothelial inflammatory processes are the initiator of atherosclerosis. Bestrophin 3 (Best-3) is involved in the regulation of cell proliferation, apoptosis and differentiation of a variety of physiological functions, but its function in cardiovascular system remains unclear. In this study, we investigated the effect of Best-3 on endothelial inflammation. We first demonstrated that Best-3 is expressed in endothelial cells and decreased after tumor necrosis factor-α (TNFα) challenge. Overexpression of Best-3 significantly attenuated TNFα-induced expression of adhesion molecules and chemokines, and subsequently inhibited the adhesion of monocytes to human umbilical vein endothelial cells (HUVECs). Conversely, knockdown of Best-3 with siRNA resulted in an enhancement on TNFα-induced expression of adhesion molecules and chemokines and adhesion of monocytes to HUVECs. Furthermore, overexpression of Best-3 with adenovirus dramatically ameliorated inflammatory response in TNFα-injected mice. Mechanistically, we found up-regulation of Best-3 inhibited TNFα-induced IKKβ and IκBα phosphorylation, IκBα degradation and NF-κB translocation. Our results demonstrated that Best-3 is an endogenous inhibitor of NF-κB signaling pathway in endothelial cells, suggesting that forced Best-3 expression may be a novel approach for the treatment of vascular inflammatory diseases.

## Introduction

Vascular inflammation is associated with accelerated many cardiovascular diseases, including atherosclerosis, hypertension and diabetes [1], [2], [3]. Endothelial dysfunction is strongly considered as a key step in the initiation and progression of vascular inflammation [4]. Multiple proinflammatory molecules, such as tumor necrosis factor α (TNFα), induce endothelial cells activation to increase the expression of adhesion molecules and many chemokines [5]. An increase in the various adhesion molecules, including intercellular adhesion molecule-1 (ICAM-1), vascular cell adhesion molecule-1 (VCAM-1), and endothelial selectin (E-selectin), as well as various chemokines and proinflammatory cytokines such as monocyte chemoattractant protein (MCP)-1, interleukin (IL)-1β and IL-8, assists recruitment of activated inflammatory cells to vascular lesions and migration into to the sub-endothelial region [6], resulting in the onset and development of vascular inflammatory diseases.

Activated NF-κB has been identified upon inflammatory stimulation, and a variety of adhesion molecules and chemokines (e.g., VCAM-1, E-selectin, ICAM-1, IL-8) have been reported to be the direct targets of NF-κB [3], [7], [8]. On stimulation, IKK (inhibitor of NF-κB) β is phosphorylated, which results in IκBα phosphorylation. Phosphorylated IκBα undergoes ubiquitination and then degradation, which activates NF-κB pathway. Once activated, NF-κB transcription factors p65 and p50 translocate from the cytoplasm to the nucleus, and subsequently regulate the transcription and expression of target genes [7], [8], [9], [10]. Although modulation of NF-κB signaling pathway has been well defined an important way for the prevention and treatment of inflammatory diseases, possible regulators remain enigmatic.

Bestrophins (Best) were first found by genetic linkage of human Best-1 to a juvenile form of macular degeneration called Best vitelliform macular dystrophy (“Best disease”) [11], and have been proposed to be a regulator of Ca^2+^-activated Cl^−^ channels in different kinds of cells [12], [13]. Three isoforms of Best have been characterized as Best-1, Best-2 and Best-3. Best-1 is mainly localized in the basolateral membrane of the retinal pigment epithelial, and mutations in Best-1 are also responsible for several other forms of retinopathy, including adult-onset vitelliform macular dystrophy [14], Bull’s eye maculopathy [15], and autosomal dominant vitreoretinochoroidopathy [16]. Best-2 is primarily expressed in nonpigmented epithelium which regulates the formation of aqueous to generate intraocular pressure [17], [18], and Best-3 is ubiquitously distributed. However, the specific function of Best-3 is poorly understood. Recent researches showed the role of mammalian Best-3 as a Ca^2+^-activated Cl^−^ channel in cardiac and vascular smooth muscle cells [19], [20]. Moreover, Jiang et al. reported Best-3 could inhibit H_2_O_2_-induced apoptosis in basilar artery smooth muscle cells [21]. Apart from these publications, there is no more information related to Best-3 function in cardiovascular system. In particular, although previous studies have evidenced that Best-3 is expressed in the heart and smooth muscle cells, the expression pattern and the functional role of Best-3 in endothelium remain obscure.

Interestingly, we found Best-3 is abundantly expressed in endothelial cells and decreased after TNFα stimulation. Therefore, in the present study, we aimed to investigate the role of Best-3 on TNFα-induced endothelial inflammatory response and the underlying molecular mechanisms. Our work reveals a novel role of Best-3 in the pathogenesis of endothelial inflammation, suggesting Best-3 may be a new strategy to prevent inflammatory diseases.

## Materials and Methods

### Ethics Statement

The prior approval was obtained for human umbilical cord from the Medical Ethical Committee, Renji Hospital, Shanghai Jiaotong University School of Medicine. Umbilical cord was collected after written informed consent from the mother after full-term pregnancies in accordance with the Declaration of Helsinki. Animal experiments were approved by the Committee on the Ethics of Animal Experiments of the Shanghai Jiaotong University School of Medicine and were in accordance with the Shanghai Jiaotong University School of Medicine guidelines for the ethical care of animals.

### Animals and Acute Inflammatory Model

C57BL/6 mice were purchased from the Jackson Laboratory (Bar Harbor, ME, USA) and housed on a 12∶12 h light/dark cycle with free access to water and diet. The acute inflammatory model was established on the basis of previous publication [22]. Mouse Best-3 adenovirus was designed and purchased from Sunbio Medical Biotechnology (Shanghai, China). At the age of 8 weeks, the mice were injected with Ad-Best-3 (10^9^ pfu/mouse) or Lacz (10^9^ pfu/mouse) via tail vein for 1 week, and then were intraperitoneally injected with 30 µg/kg TNFα (Sigma Chemical Co., St. Louis, USA) for another 3 days.

### Cell Culture

Human umbilical vein endothelial cells (HUVECs) were isolated from human umbilical cords and cultured as previously described [23]. Briefly, HUVECs were harvested with 0.125% trypsin containing 0.01% EDTA, and then the cells were cultured in EBM-2 medium (Gibco, Carlsbad, USA) with supplemented growth factors according to the manufacturer’s instructions in a humidified atmosphere of 5% CO_2_ at 37°C and were used in experiments at passage 4 to 6.

For isolation of mouse aortic endothelial cells (MAECs), aorta was collected from mouse under anaesthesia with sodium pentobarbital (50 mg/kg, i.p.). After fat and connective tissue were carefully cleaned, the aorta was cut into 3 mm long sections and placed on matrigel pre-coated plates and cultured in DMEM/F12 medium (Gibco) with supplemented growth factors at 37°C. After 5–7 days, MAECs began to migrate from the aortic segments. When reaching confluence, TNFα (10 ng/ml) was added again to MAECs to potential its effect. 24 h later, the cells were used for western blot, RT-PCR, monocyte adhesion assay, or ELISA.

THP-1 cells were cultured in RPMI 1640 culture medium (Gibco) containing 10% fetal calf serum at 37°C in a humidified incubator of 5% CO_2_ at 37°C.

### Small Interfering RNA Transfection

Stealth human Best-3 (GeneBank Accession No. NM_001282613) siRNA duplex oligoribonucleotides 5′-UUCACUACCAGAGUAACGU-3′ was obtained from Qiagen (CA, USA). The siRNA were transfected transiently with Hiperfect Transfection Reagent (Qiagen) according to the manufacturer’s instructions, and a negative siRNA (Neg. RNA) was used as a control. In this study, HUVECs were transfected with 40 nM Neg. RNA or siRNA for 48 h in the presence or absence of TNFα (10 ng/ml).

### Adenovirus Infection

Human Best-3 adenovirus (Ad-Best-3) was purchased from Sunbio Medical Biotechnology. Briefly, full-length cDNA of human Best-3 was cloned into plasmid pCMV-Tag2 between BamH1 and Xho1 restriction sites, and this recombinant plasmid was packaged into adenovirus. An adenovirus bearing LacZ was obtained from Clontech (CA, USA). HUVECs were seeded in 6-well plate in complete medium overnight. After washing with PBS, the cells were cultured in 700 µl normal medium without serum. The adenovirus vectors (MOI = 25, 50, 100) were diluted in 100 µl normal medium, and then were added to the cells gently. After 6 h, the cells were transferred into complete medium and cultured for 48 h. In this study, HUVECs were infected with 50 MOI Lacz or Ad-Best-3 for 48 h prior to TNFα incubation.

### Western Blot Analysis

Thoracic aorta homogenates or cell extracts were applied for western blot analysis as previously described [23]. In briefly, tissues or cells were washed with cold PBS three times, and then lysed in RIPA buffer (Beyotime, Jiangsu, China) containing protease and phosphatase inhibitor cocktail (Merck, Darmstadt, Germany). For nuclear p65 and p50 detection, nuclear proteins were extracted using a Nuclear/Cytosol Fractionation Kit (BioVision, Milpitas, CA, USA) according to the manufacturer’s instructions. Equal amount of proteins, determined using Bradford assay (Bio-Rad Laboratories, Hercules, USA) were separated by SDS-polyacrylamide gel electrophoresis and transferred to nitrocellulose membranes (Millipore, Billerica, USA). After blocking, the membranes were incubated with the following primary antibodies at 4°C overnight: p65, p50, p-IκBα, IκBα and p-IKKβ (diluted 1∶1000; Cell Signaling Technology, MA, USA); Best-3, ICAM-1 and VCAM-1 (diluted 1∶500), Lamin B and GAPDH (diluted 1∶1000) (Santa Cruz, CA, USA). After washing and incubation with secondary antibodies including HRP-conjugated anti-rabbit or anti-goat (diluted 1∶1000; Cell Signaling Technology) for 1 h, membranes were visualized with ECL system (Beyotime). Image quantification was performed using ImageJ software (NIH, Maryland, USA).

### Real-time Quantitative PCR Analysis

Total RNA from HUVECs or MAECs or thoracic aorta was isolated using RNeasy Micro Kit (Qiagen). Reverse transcription was performed using the ReverTra ACE qPCR RT Kit (Toyobo, Osaka, Japan). Real-time PCR using SYBR Green PCR Master Mix reagents (Applied Biosystems, Grand Island, USA) was carried out on the Sequence Detector System software version 2.1 (Applied Biosystems Prism 7300). The PCR procedure was as follows: 94°C for 4 min; 95°C for 10 s, 60°C for 30 s; 72°C for 20 s. Relative expression was determined using GAPDH as an internal control and reported as 2^−ΔΔCT^. Specific primers (Table S1 in File S1) for Best-1, Best-2, Best-3, ICAM-1, VCAM-1 and GAPDH were synthesized by Invitrogen (Grand Island, USA).

### Monocyte Adhesion Assay

HUVECs were pretreated with Ad-Best-3 or Best-3 siRNA prior to TNFα (10 ng/ml) incubation for another 24 h in 35 mm culture dishes at a density of 2×10^5^ cells/ml. After treatment, THP-1 cells were labeled with 5 µM VibrantDiO Cell-Labeling Solution (Molecular Probes, NY, USA) and added to each culture dish and allowed to adhere for 30 min at 37°C, 5% CO_2_. The dishes were gently washed to remove non-adherent cells twice and the adherent cells were visualized using confocal microscopy (LSM710, ZEISS, München, Germany).

### Enzyme Linked Immunosorbent Assay (ELISA)

Cell culture supernatant and serum were analyzed for human or mouse ICAM-1, VCAM-1 and E-selectin using an ELISA kit (Abcam, MA, USA). The concentration of chemokines and proinflammatory cytokines in cell lysates and serum were determined by human or mouse MCP-1, IL-1β and IL-8 ELISA Kit (R&D Systems, Minnesota, USA). All measurements were performed as recommend by the manufacturer.

### Immunoprecipitation

HUVECs were transfected with Lacz or Ad-Best-3 for 48 h prior to TNFα stimulation for 30 min. Cells were lysed in RIPA buffer supplemented with protease and phosphatase inhibitor cocktail. Protein A/G agarose beads (Santa Cruz) was added to the lysate to pre-clear nonspecific binding. Equal amounts of proteins determined by Bradford assay were co-incubated with IκBα antibody and protein A/G agarose beads at 4°C overnight. The immunoprecipitates were washed four times with RIPA buffer and the immunoprecipitated proteins were analyzed by western blot using ubiqutin antibody (1∶1000 dilution; Cell Signaling Technology).

### Immunofluorescent Staining

Mouse thoracic aorta was carefully isolated, and embedded in optimal cutting temperature compound (OTC, Tissue-Tek, Sakura, Japan) and snap-frozen in liquid nitrogen. Samples were cut into 8 µm longitudinal cryosections for immunofluorescence staining. Frozen tissue sections were fixed with 4% paraformaldehyde and washed with PBS containing 0.1% Triton X-100 for 3 times. Nonspecific binding was blocked by 5% rabbit serum solution for 1 h at room temperature. After blocking, the sections were incubated with primary antibodies against Best-3 (diluted 1∶100, goat anti-rabbit), CD31 (diluted 1∶200, donkey anti-goat; Abcam), ICAM-1 (diluted 1∶200, donkey anti-goat) or VCAM-1 (diluted 1∶200, donkey anti-goat) at 4°C overnight. Sections were then washed with PBS, co-incubated with the secondary antibodies (Cy3-conjugated anti-goat or FITC-conjugated anti-rabbit, diluted 1∶200; Beyotime) at room temperature for 1 h. The nucleus was stained with Hoechst 33258. The sections were observed using a confocal microscopy (LSM710, ZEISS).

### Statistical Analysis

All data were expressed as mean value ± standard error of mean (SEM). The one-way ANOVA followed by Bonferroni multiple comparison post hoc test with 95% CI was used by SPSS17.0 statistical software (SPSS Inc., IL, USA). P value of less than 0.05 was considered statistically significant.

## Results

### TNFα Impaired Endogenous Best-3 Expression in Endothelium

**Figure 1:**
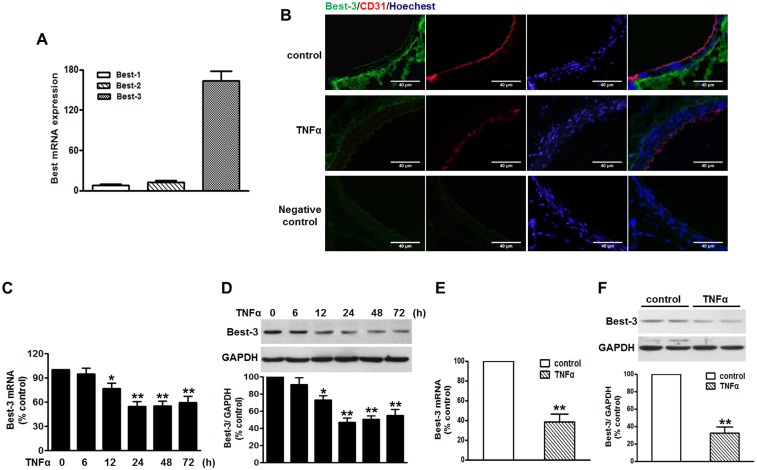
TNFα decreased Best-3 expression in endothelium. (**A**) quantitative PCR analysis of Best-1, Best-2 and Best-3 mRNA expression in HUVECs. n = 6. (**B**) immunofluorescent staining of Best-3 (green) and CD31 (red) and Hoechst (blue) in thoracic aorta of mice after TNFα challenge for 3 days. n = 4–6. Scale bars, 40 µm. (**C, D**) HUVECs were stimulated with TNFα (10 ng/ml) for different times as indicated. Quantitative PCR (C) and western blot (D) results show a time-dependent decrease of Best-3 expression. *P<0.05, **P<0.01 vs. untreated group, n = 5. (**E, F**) quantitative PCR (E) and western blot (F) analysis of Best-3 expression in MAECs isolated from mice after treatment mentioned in method section, respectively. **P<0.01 vs. control group, n = 8.

First of all, we analyzed the expression pattern of Best family (Best1, Best-2 and Best-3) in HUVECs. Quantitative RT-PCR analysis showed that Best-3 expression was prominent in HUVECs, whereas the expression of other members was too faint to be detected (Figure 1A), indicating that Best-3 may mediate specific functions in endothelial cells. TNFα is one of the most important proinflammatory molecules that increases the expression of adhesion molecules and induces endothelial inflammatory response [24]. Here, we examined the effect of TNFα on Best-3 expression in endothelia cells. Immunofluorescent staining showed that Best-3 was expressed in endothelium that expressed endothelial cells marker CD31. Although Best-3 staining displayed a dominant signal in the media layer and was faint in endothelium, negative control staining only with fluorescent-labeled secondary antibodies, without primary antibodies, revealed no significant immunofluorescence, indicating it was not a non-specific fluorescence in endothelium. Of note, injection with TNFα (30 µg/kg) translated into an apparent damage of the endothelium and an impairment of staining of CD31. Interestingly, overall Best-3 expression was dramatically decreased in TNFα-injected mice (Figure 1B). Consistent with the previous data, western blot also showed Best-3 expression was reduced in aortas which were stripped or not stripped from endothelium following TNFα stimulation (Figure S1A and S1B in File S1), suggesting it may be not an endothelial-specific event. Therefore, to further confirm the reduction of Best-3 expression in endothelial cells, we determined the mRNA and protein levels of Best-3 not only in HUVECs but also in MAECs. Expectedly, Best-3 expression was decreased significantly after TNFα treatment in the both endothelial cells (Figure 1C, 1D, 1E and 1F). Moreover, CD31 expression in MAECs remained unchanged following TNFα stimulation, indicating the same level of purity in both isolations (Figure S1C in File S1).

### Best-3 Inhibited TNFα-Induced Inflammatory Response in Endothelial Cells

**Figure 2:**
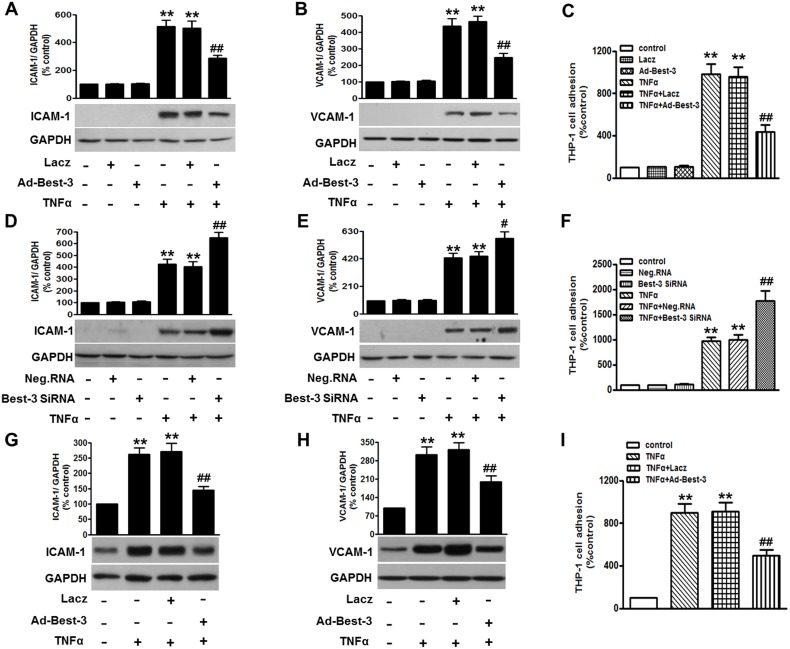
Best-3 ameliorated TNFα-induced inflammatory response in endothelial cells. (**A, B**) HUVECs were transfected with Lacz or Ad-Best-3 for 48 h prior to TNFα treatment for 24 h. ICAM-1 (A) and VCAM-1 (B) were examined by western blot, respectively. (**C**) after treatment mentioned in (A, B), adhesion of VibrantDiO-labeled THP-1 to HUVECs were analyzed. (**D, E**) HUVECs were transfected with negative siRNA (Neg. RNA) or Best-3 siRNA for 48 h prior to TNFα incubation. ICAM-1 (D) and VCAM-1 (E) were detected by western blot, respectively. (**F**) after treatment mentioned in (D, E), adhesion of THP-1 to HUVECs was analyzed. (**G, H**) western blot detection of ICAM-1 (G) and VCAM-1 (H) expressions in MAECs isolated form mice after treatment mentioned in method section. (**I**) adhesion of THP-1 to MAECs was analyzed. All data are presented as mean ± SEM. **P<0.01 vs. control, ^#^P<0.05, ^##^P<0.01 vs. TNFα alone, n = 6.

Based on the decrease of Best-3 expression after TNFα challenge in endothelial cells, we speculated that Best-3 may be related to endothelial inflammation induced by TNFα. To verify this assumption, we used adenovirus to overexpress Best-3 or small interfering RNA (siRNA) to knockdown Best-3 in HUVECs. The successful overexpression and knockdown of Best-3 were confirmed by western blot (Figure S2A and S2B in File S1). We found neither Best-3 overexpression nor knockdown had significant effects on basal ICAM-1 and VCAM-1 expressions. However, overexpression of Best-3 significantly inhibited TNFα-induced expression of ICAM-1 and VCAM-1 and adhesion of THP-1 cells to HUVECs (Figure 2A, 2B and 2C) (Figure S2C, S2D and S3A in File S1). Expectedly, Best-3 siRNA dramatically enhanced TNFα-induced expression of adhesion molecules (Figure 2D, 2E and 2F) (Figure S2E, S2F and S3B in File S1). Moreover, we determined the levels of the inflammatory mediators of ICAM-1, VCAM-1, endothelial activation marker-E-selectin, MCP-1, IL-1β and IL-8 by ELISA assay. As a result, up-regulation of Best-3 markedly inhibited TNFα-induced secretion of these inflammatory mediators, and inverse results were obtained in Best-3 siRNA-treated cells (Figure S4A and S4B in File S1).

To further validate the protective role of Best-3 overexpression in vascular inflammation, we *in vivo* established an acute inflammatory model with a gene approach as schematically illustrated (Figure S5A in File S1). In these experiments, wild-type C57BL/6 mice were injected by tail vein with 10^9^ plaque-forming units of Ad-Best-3 (n = 24) or an adenoviral vector Lacz (n = 24) in the presence of TNFα. The infection efficiency of Best-3 adenovirus was confirmed in aortas and MAECs by western blot (Figure S5B and S5C in File S1). In MAECs isolated from TNFα-treated Ad-Best-3-infected mice, the increases of the expression of ICAM-1 and VCAM-1 and adhesion of THP-1 cells to MAECs after TNFα treatment were all significantly attenuated (Figure 2G, 2H and 2I) (Figure S3C, S5D and S5E in File S1). Lacz infection did not alter the adhesion molecules expression modulation induced by TNFα. In addition, we found Ad-Best-3 infection also markedly suppressed TNFα-induced secretion of ICAM-1, VCAM-1, E-selectin, MCP-1, IL-1β and IL-8 in MAECs (Figure S4C in File S1), similar to what was observed in HUVECs. These findings suggest that Best-3 plays a protective role in TNFα-induced endothelial inflammation.

### Best-3 Ameliorated TNFα-Induced Inflammatory Response *in vivo*

**Figure 3:**
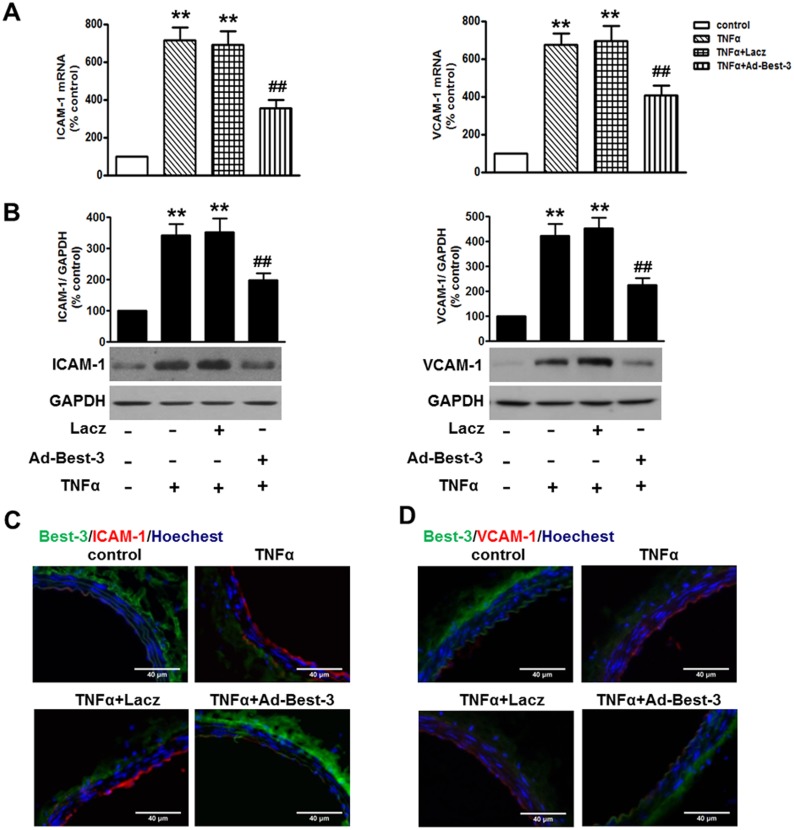
Reduced inflammatory response in Ad-Best-3-infected mice. C57BL/6 mice were infected with Ad-Best-3 (10^9^ pfu/mouse) or Lacz (10^9^ pfu/mouse) for 1 week in the presence of TNFα (30 µg/kg) for another 3 days. (**A. B**) the expression of ICAM-1 and VCAM-1 in the aorta was analyzed by quantitative PCR (A, upper panel) and western blot (B, middle panel), respectively. **P<0.01 vs. control, ^##^P<0.01 vs. TNFα alone, n = 7. (**C, D**) immunofluorescent staining of Best-3 (green) and ICAM-1 (red) and Hoechst (blue) (C), or Best-3 (green) and VCAM-1 (red) and Hoechst (blue) (D) in thoracic aorta of mice, respectively. Scale bars, 40 µm. n = 4–6.

In line with the results *in vitro*, our results showed TNFα-induced increase of the expression of ICAM-1 and VCAM-1 both on mRNA and protein levels in thoracic aorta were all obviously alleviated in Ad-Best-3-infected mice (Figure 3A and 3B). Immunofluorescent staining also revealed attenuated ICAM-1 and VCAM-1 expression in TNFα-injected Ad-Best-3-infected mice, an effect that was not observed in Lacz-infected mice (Figure 3C and 3D). Moreover, the secretion level of ICAM-1, VCAM-1, E-selectin, MCP-1, IL-1β and IL-8 in the serum harvested from TNFα-injected Ad-Best-3-infected mice was decreased compared to those in TNFα-injected mice (Figure S6 in File S1).

### Best-3 Inhibited TNFα-Induced NF-κB Activation

**Figure 4:**
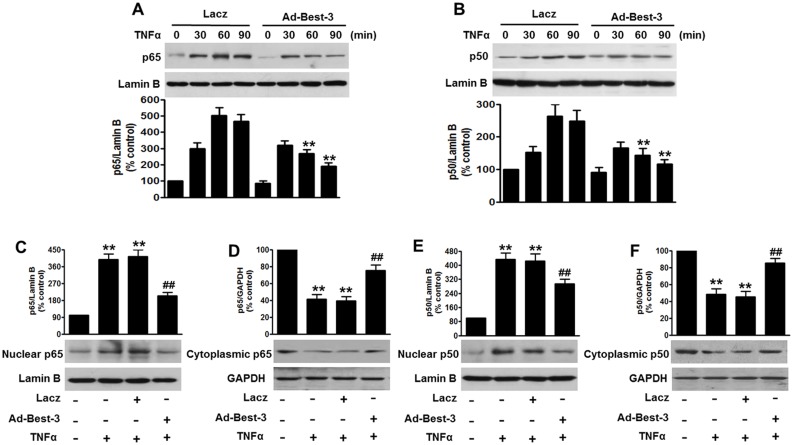
Best-3 repressed TNFα-induced NF-κB activation in endothelial cells. (**A, B**) HUVECs were infected with Lacz or Ad-Best-3 for 48 h, and then incubated with TNFα (10 ng/ml) for different times as indicated. Nuclear fractions were isolated and detected by western blot using p65 (A) and p50 (B) antibodies. **P<0.01 vs. similarity treated control, n = 4. (**C–F**) nuclear and cytoplasmic fractions of MAECs isolated from mice after treatment mentioned in method section were analyzed by western blot to detect the expressions of p65 (C, D) and p50 (E, F). **P<0.01 vs. control, ^##^P<0.01 vs. TNFα alone, n = 6.

In response to proinflammatory stimulation, NF-κB pathway is involved in the inflammatory responses in endothelial cells [25]. To investigate whether Best-3 regulates the extent of NF-κB activation, HUVECs were pretreated with adenoviral vector Lacz or Ad-Best-3 for 48 h, and then incubated with TNFα over a range of indicated time points. Cell lysates were western blotted for p65 and p50 to analysis NF-κB nuclear accumulation. As shown in Figure 4A, nuclear translocation of p65 was triggered within 30 min and reached a maximum at 60 min in Lacz-treated cells. Although Ad-Best-3-treated HUVECs showed peak p65 translocation within 30 min, p65 nuclear accumulation was obviously less than Lacz-treated cells and then was declined rapidly over the time points tested. Moreover, nuclear translocation of p50 remained similar change pattern to those observed in p65 translocation (Figure 4B), indicating Best-3 mainly through reducing p65 and p50 nuclear accumulation to regulate negatively NF-κB activation.

Translocation of NF-κB from the cytoplasm to the nucleus is an essential step for the activation of NF-κB target genes [26], [27], which is critical for vascular inflammation *in vivo*. Here, we explored the effect of Best-3 on p65 and p50 protein levels in cytoplasmic and nuclear fractions of MAECs. In mice not treated with TNFα, the majority of p65 and p50 remained in cytoplasmic fractions of MAECs. TNFα dramatically enhanced p65 and p50 nuclear accumulation. However, pre-infection with Ad-Best-3 demonstrated reduced p65 and p50 expression by 48% and 33% in the nuclear fraction, respectively, whereas the cytoplasmic fraction had increased p65 and p50 expression by 45% and 41%, respectively. In addition, the activation of NF-κB induced by TNFα was not significantly altered in TNFα-treated Lacz-infected mice (Figure 4C, 4D, 4E and 4F). Collectively, these data suggest that the suppression of NF-κB activation underlies the anti-inflammation effect of Best-3 in endothelial cells.

### Best-3 Suppressed IKKβ/IκBα Signaling in Endothelial Cells

**Figure 5:**
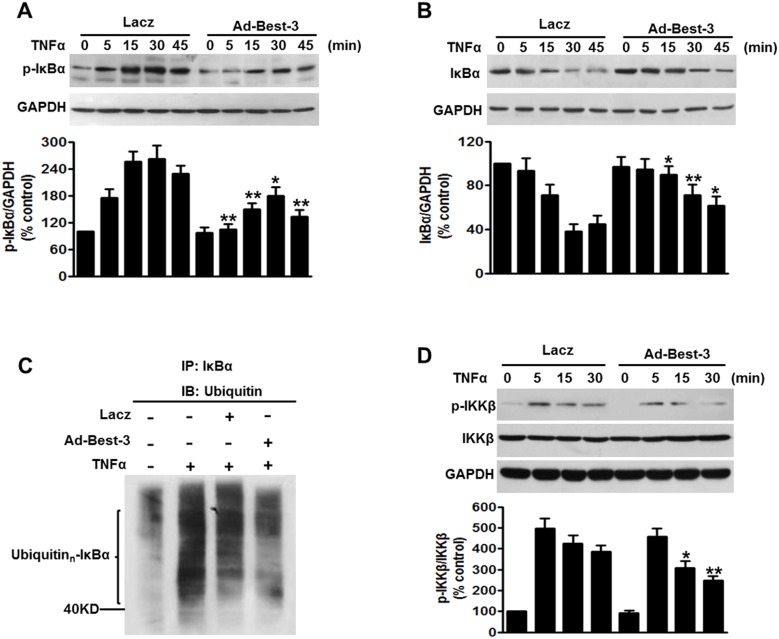
Best-3 suppressed IKKβ/IκBα pathway to inhibit inflammation. (**A, B**) HUVECs were infected with Lacz or Ad-Best-3 for 48 h, and then incubated with TNFα (10 ng/ml) for different times as indicated. Cell lysates were subjected to western blot analysis using p-IκBα (A) and IκBα (B) antibodies. *P<0.05, **P<0.01 vs. similarity treated control, n = 4. (**C**) western blot analysis of ubiquitinated IκBα in HUVECs treated with Lazc or Ad-Best-3 in the presence of TNFα for 30 min. Cell lysates were immunoprecipitated with IκBα antibody and immunoprecipitated proteins were blotted with ubiquitin antibody to reveal ubiquitination of IκBα. (**D**) western blot analysis of p-IKKβ and IKKβ of HUVECs treated with Lacz or Ad-Best-3 for 48 h in the presence of TNFα for different times as indicated. *P<0.05, **P<0.01 vs. similarity treated control, n = 4.

To further explore the mechanism by which Best-3 inhibits NF-κB activation, we investigated the effect of Best-3 on NF-κB upstream signaling pathway, IKKβ/IκBα. Our results showed TNFα induced phosphorylation of IκBα at 5 min in Lacz-treated cells, which maintained this induction over a range of indicated time points. However, overexpression of Best-3 significantly inhibited TNFα-induced phosphorylation of IκBα, which delayed the peak of IκBα phosphorylation at 30 min (Figure 5A). In addition, TNFα induced decrease of IκBα expression from 15 min in Lacz-treated cells and reached a maximum at 30 min. Nevertheless, up-regulation of Best-3 remarkably antagonized against the degradation of IκBα induced by TNFα, which triggered form 30 min and reached a maximum at 45 min (Figure 5B). This was further supported by the result of IκBα ubiquitination (Figure 5C). Notably, there was no significant difference in the onset of IKKβ phosphorylation between Lacz and Ad-Best-3 group. However, IKKβ phosphorylation was obviously declined after 5 min in Ad-Best-3-treated cells, compared with those from Lacz-treated cells (Figure 5D). These results suggest that Best-3 functions as a negative regulator of IKKα/IκBα signaling via inhibiting IκBα degradation and IκBα and IKKβ phosphorylation in endothelial inflammation.

## Discussion

It has been reported that patients with inflammatory diseases present sustained endothelial cells activation and subsequently result in endothelial dysfunction, often in the earliest period of cardiovascular disease [5], [28]. Therefore, mechanisms linking inflammation and cardiovascular diseases may be best understood at the level of the endothelium. In the present study, we demonstrated Best-3 may be a critical regulator in endothelial inflammatory response. In support, using up- and down-regulation of Best-3 expression approaches in HUVECs, we evidenced that Best-3 inhibited TNFα-induced NF-κB activation by directly repressing nuclear accumulation of p65 and p50. Further studies showed that Best-3 may target the upstream of NF-κB signaling pathway, IKKβ/IκBα, and thereby inhibited NF-κB-dependent genes expressions associated with inflammatory diseases, including cell adhesion molecules and other key chemokines *in vitro*. Importantly, systemic infection of Ad-Best-3 revealed an inhibition on NF-κB nuclear translocation and subsequently significantly ameliorated TNFα-induced inflammatory response *in vivo*.

Bestrophins (Best), a newly identified family of Cl^−^ channels, function as regulators of voltage-gated Ca^2+^ channels. Some Best are activated by increases in intracellular Ca^2+^ concentration, but whether Best are the molecular candidates of Ca^2+^-activated Cl^−^ channels remains doubtful [20], [29]. For a long time researchers mainly focused on this controversial topic, but the exact function of Best proteins is poorly understood. Best-1 and Best-2 have been identified mainly both in human epithelial cells, whereas Best-3 is widely expressed in a variety of tissues. Recent accumulating evidence has demonstrated that Best is involved in proliferation in colonic cancer cells [30], apoptosis in vascular smooth muscle cells [21], cell death in renal epithelial cells [31] and vasomotion in rat mesenteric small arteries [32]. However, their expression profile and function in cardiovascular system remain elusive. The expression of Best-3 has been detected in endothelial layer of rat mesenteric small arteries [32]. Consistent with this study, we found Best-3 is highly expressed in HUVECs based on quantitative PCR, but the endogenous expression of Best-1 and Best-2 is very faint. Furthermore, we confirmed this by immunofluorescent staining of Best-3 and endothelial cells marker CD31, and demonstrated that Best-3 is located in endothelium of thoracic aorta, indicating Best-3 may play a functional role in regulating endothelial homeostasis. The vascular endothelium has been suggested to be a target of TNFα. In endothelial cells, TNFα induces the expression of genes associated with inflammation, which appears to be a classic inflammatory model [28]. Interestingly, not only by quantitative PCR and western blot *in vitro* and *in vivo* but also by immunofluorescent staining, we noticed the expression of Best-3 was significantly decreased after TNFα challenge. These results strongly suggest that Best-3 is involved in endothelial inflammation.

The increased expression of adhesion molecules and chemokines is the earliest important events during the pathogenesis of inflammation [33], [34]. In our study, we choose several representative and critical chemokines to analyze. ICAM-1, VCAM-1 and E-selectin are adhesion molecules, which recruits immune cells to the vascular endothelium, a characteristic of inflammation [33], [35]. Moreover, some key chemokines and proinflammatory cytokines such as MCP-1, IL-1β and IL-8 have been reported to play a crucial role in inflammatory injury [36], [37], [38]. We have shown that atorvastatin significantly decreased MCP-1 expression level in atherosclerotic plaques, and thereby improved plaque stability [38]. Here, our results showed that knockdown of Best-3 enhanced the expression of ICAM-1 and VCAM-1, as well as the secretion of E-selectin, MCP-1, IL-1β and IL-8. In contrast, overexpression of Best-3 significantly inhibited the expression of these inflammatory factors. Agreement with the results *in vitro*, in MAECs and aorta from systemic Ad-Best-3 infection mice, TNFα-induced endothelial inflammation was remarkably attenuated.

NF-κB is an important mediator of inflammatory disorder that can be rapidly activated by a variety of inflammatory stimulation, such as TNFα and LPS [24], [39], [40]. Our previous study demonstrated that the involvement of the JAK/STAT signaling pathway in the occurrence and development of myocardial infarction is closely correlated with its promotion on NF-κB activation and TNFα expression [41]. Here, we found overexpression of Best-3 inhibited TNFα-induced IKKβ and IκBα phosphorylation, blocked TNFα-induced ubiquitination and subsequent degradation of IκBα, and suppressed the nuclear translocation of p65 and p50. Importantly, we also demonstrated that systemic infection of Ad-Best-3 *in vivo* drastically reduced nuclear accumulation of p65 and p50. Our present study suggested Best-3 is an inhibitor of the NF-κB activation, which had not been shown before.

In conclusion, our study demonstrated Best-3 ameliorated TNFα-induced inflammation by inhibiting NF-κB activation, and revealed an anti-inflammatory function of Best-3 *in vitro* and *in vivo*. However, further studies are needed to elucidate the exact mechanism by which Best-3 inhibited NF-κB signaling pathway, and to identify whether Best-3 can be exploited as a potential therapeutic target for inflammatory diseases.

## Supporting Information

File S1 **File contains Table S1 and Figures S1–S6.**
(DOC)
