# Supplementary material for: Bestrophin 3 Ameliorates TNFα-Induced Inflammation by Inhibiting NF-κB Activation in Endothelial Cells
Source: PLoS One. 2014 Oct 20;9(10):e111093. doi: 10.1371/journal.pone.0111093 (PMC4203846; doi:10.1371/journal.pone.0111093)
Supplement: File S1 — File contains Table S1 and Figures S1–S6. (DOC) [file pone.0111093.s001.doc]

**Bestrophin 3 Ameliorates TNFα-Induced Inflammation by Inhibiting NF-κB Activation in Endothelial Cells**

Wei Song^1^*, Zhen Yang^2^*, Ben He^1^

^1^Department of Cardiology, Renji Hospital, School of Medicine, Shanghai Jiaotong University, Shanghai, 200127, China. ^2^Department of Hypertension and Vascular Disease, The First Affiliated Hospital of Sun Yat-Sen University, Guangzhou, 510080, China.

Correspondence to:

Ben He, Department of Cardiology, Renji Hospital, School of Medicine, Shanghai Jiaotong University, 1630 Dongfang Rd, Shanghai, 200127, People’s Republic of China.

Tel：86-021-58752345

E-mail: rjheben@21cn.com

*These authors contribute equally to this work

**ONLINE SUPPLEMENTAL MATERIALS**

**Supplemental Tables**

**Table S1**. Sequence of primers used in quantitative real-time PCR

| Gene | Sequence (forward) | Sequence (reverse) |
| --- | --- | --- |
| Best-1 (human) | 5’ atgaccatcacttacacaagccc 3’ | 5’ ctcgtactggttccagcggg 3’ |
| Best-2 (human) | 5’ agcctcgtgtgcctttgtaa 3’ | 5’ gagtctgcaagaactcccgc 3’ |
| Best-3 (human) | 5’ tggtagtgaacccatggtgg 3’ | 5’ gagcgaaagatgagcaggga 3’ |
| Best-3 (mouse) | 5’ aaaggctacgtaggacatga 3’ | 5’ gaaaggacggtatgcagtag 3’ |
| ICAM-1 (human) | 5’ gcagacagtgaccatctacagctt 3’ | 5’ cttctgagacctctggcttcgt 3’ |
| ICAM-1 (mouse) | 5’ ccggtcctgaccctgagcca 3’ | 5’ attggacctgcggggtgggt 3’ |
| VCAM-1 (human) | 5’ ggcaggctgtaaaagaattgca 3’ | 5’ gtcatggtcacagagccacctt 3’ |
| VCAM-1 (mouse) | 5’ gacaggcgacatggtattaaag 3’ | 5’ gccaacttcagtcttaga 3’ |
| GAPDH (human) | 5’ agctgaacgggaagctcactgg 3’ | 5’ ggagtgggtgtcgctgtgaagtc 3’ |
| GAPDH (mouse) | 5’ cttcaccaccatggagaaggc 3’ | 5’ ggcatggactgtggtcatgag 3’ |

**Supplemental Figures**


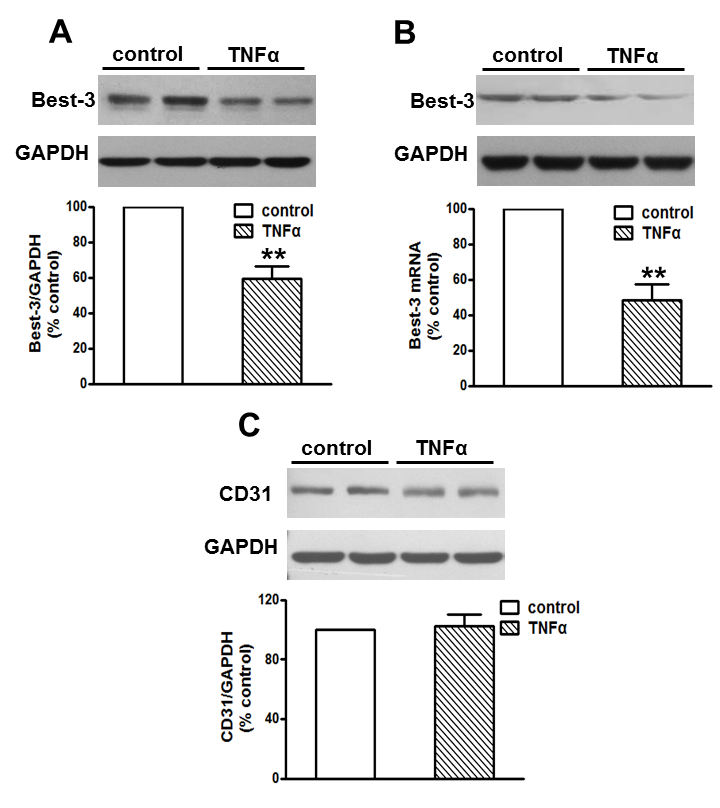
**Figure S1**

**Figure S1.** **TNFα decreased Best-3 expression in aortas. (A, B)** Best-3 expression in aortas which were stripped (A) or not stripped (B) from endothelium following TNFα stimulation was analyzed by western blot using GAPDH as an internal control. ^**^P<0.01 vs. control group, n=8. **(C)** western blot showed CD31 expression in MAECs remained unchanged following TNFα stimulation.


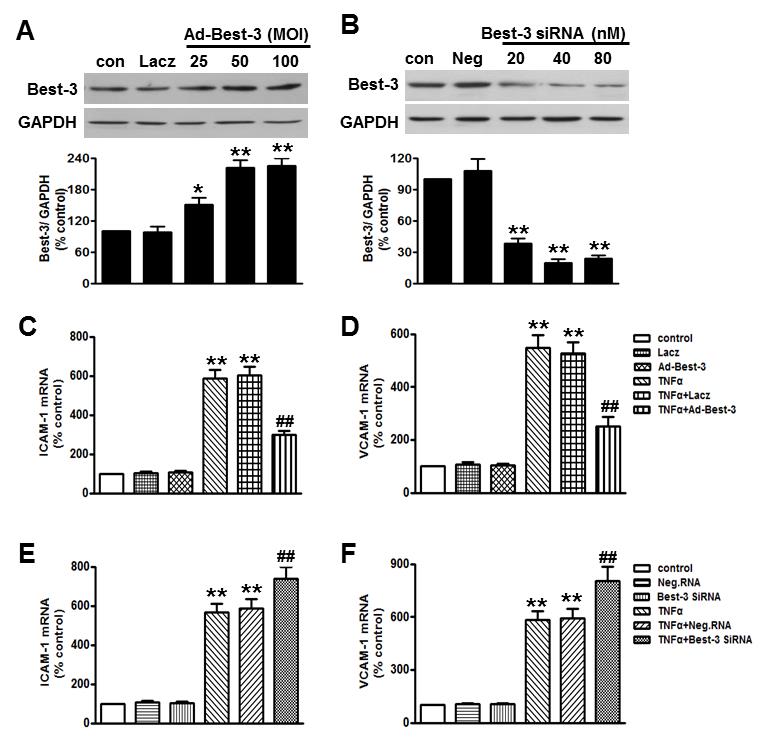
**Figure S2**

**Figure S2. Best-3 inhibited TNFα-induced ICAM-1 and VCAM-1 expression in HUVECs. (A)** cells were infected with adenovirus packing expression vector of Best-3 (Ad-Best-3, MOI=25, 50, 100) or Lacz (MOI=100) for 48 h. Expression of Best-3 was examined by western blot using GAPDH as an internal control. ^*^P<0.05, ^**^P<0.01 vs. control, n=6. **(B)** cells were transfected with Best-3 siRNA for different concentrations (20, 40, 80 nM) or negative siRNA (Neg.RNA) for 48 h. The efficiencies of siRNA were detected by western blot. ^**^P<0.01 vs. control, n=6. **(C, D)** HUVECs were transfected with Lacz or Ad-Best-3 for 48 h prior to TNFα treatment for 24 h. The mRNA levels of ICAM-1 (C) and VCAM-1 (D) were analyzed by quantitative PCR, respectively. ^**^P<0.01 vs. control, ^##^P<0.01 vs. TNFα alone, n=4. **(E, F)** HUVECs were transfected with Neg.RNA or Best-3 siRNA for 48 h prior to TNFα incubation. The mRNA levels of ICAM-1 (E) and VCAM-1 (F) were analyzed by quantitative PCR, respectively. ^**^P<0.01 vs. control, ^##^P<0.01 vs. TNFα alone, n=4.


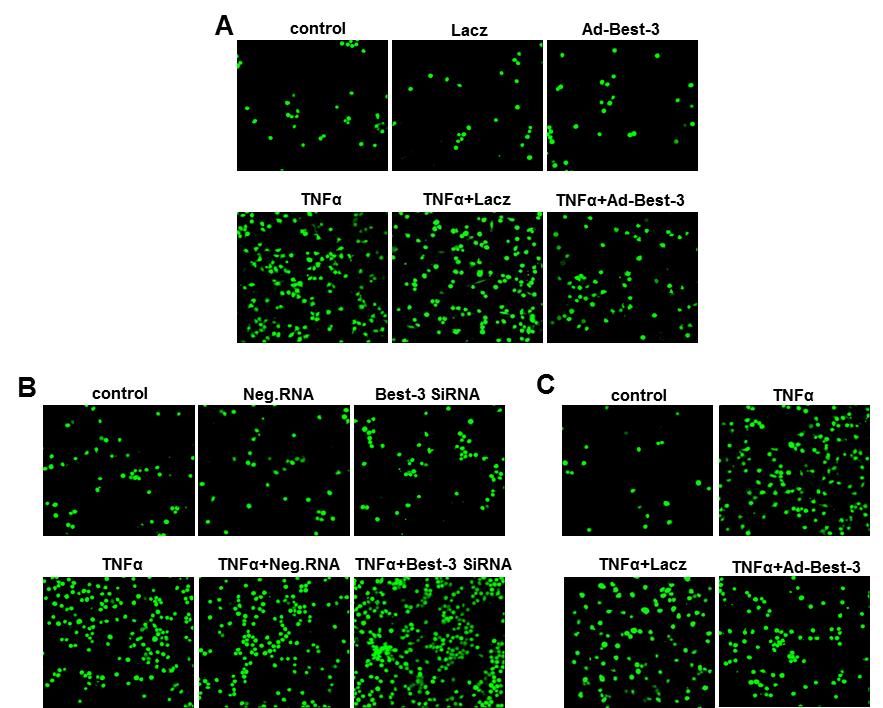
**Figure S3**

**Figure S3. Best-3 inhibited TNFα-induced adhesion of monocyte to endothelial cells. (A, B, C)** the adhesion of THP-1 cells to HUVECs infected with Lacz or Ad-Best-3 for 48 h prior to TNFα treatment for 24 h (A), or to HUVECs transfected with Neg.RNA or Best-3 siRNA for 48 h prior to TNFα incubation (B), or to MAECs isolated form mice after treatment mentioned in method section (C) was detected by fluorescence microscopy.


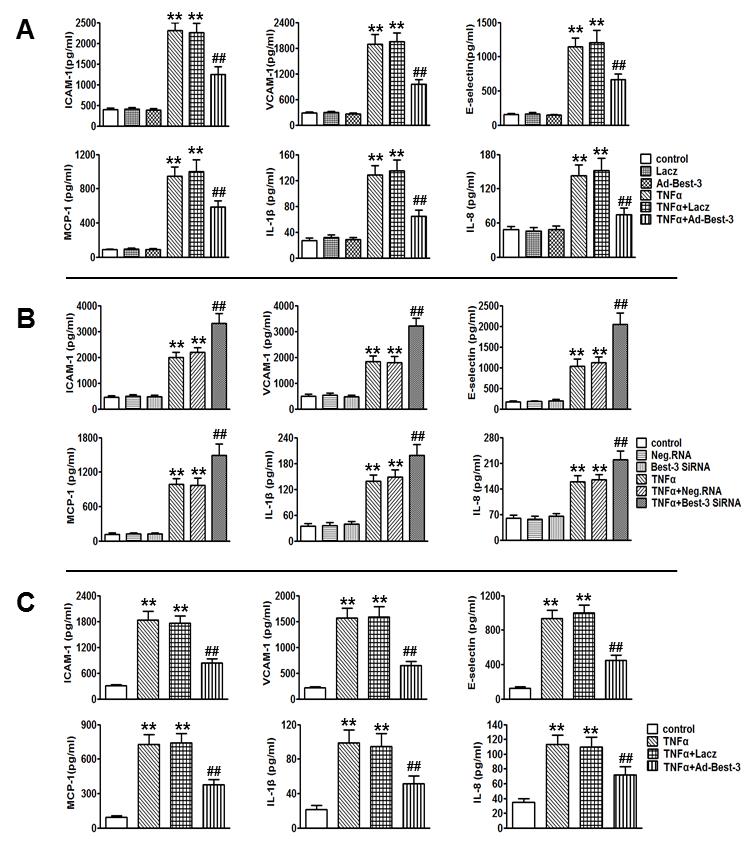
**Figure S4**

**Figure S4. Best-3 inhibited TNFα-induced secretion of inflammatory mediators in endothelial cells. (A, B, C)** the levels of ICAM-1, VCAM-1, E-selectin, MCP-1, IL-1β and IL-8 in HUVECs infected with Lacz or Ad-Best-3 for 48 h prior to TNFα treatment for 24 h (A, upper panel), or in HUVECs transfected with Neg.RNA or Best-3 siRNA for 48 h prior to TNFα incubation (B, middle panel), or in MAECs isolated form mice after treatment mentioned in method section (C, lower panel) were determined by ELISA assay. All data are presented as mean ± SEM. ^**^P<0.01 vs. control, ^##^P<0.01 vs. TNFα alone, n=6.


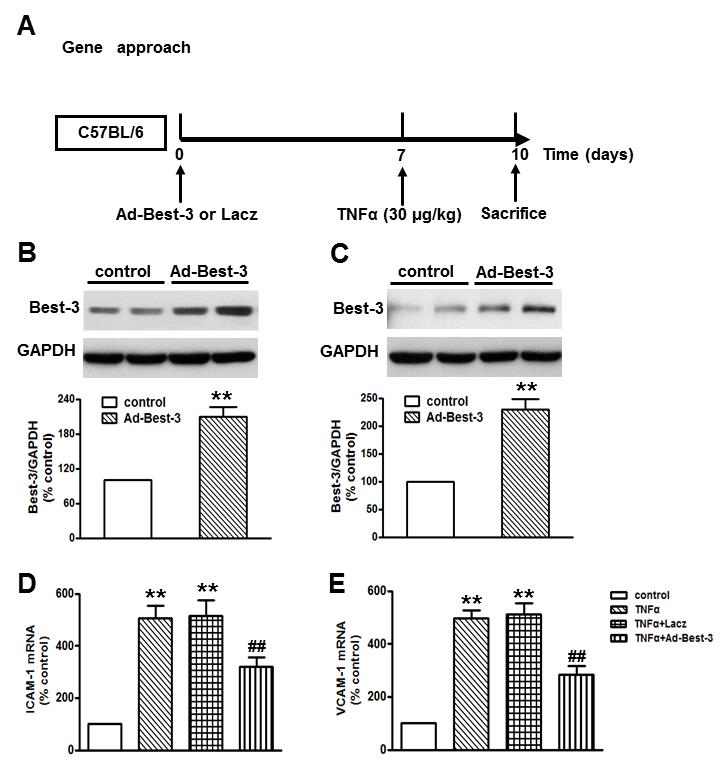
**Figure S5**

**Figure S5. Best-3 inhibited TNFα-induced ICAM-1 and VCAM-1 mRNA expression in MAECs. (A)** animal experimental design. C57BL/6 mice were injected with Lacz (10^9^ pfu/mouse) or Ad-Best-3 (10^9^ pfu/mouse) via tail vein for 1 week, and then were intraperitoneal injected with 30 µg/kg TNFα for 3 days. **(B, C)** the infection efficiency of Best-3 adenovirus was confirmed in aortas (B) and MAECs (C) by western blot. **(D, E)** the mRNA expression of ICAM-1 (D) and VCAM-1 (E) in MAECs was analyzed by quantitative PCR. ^**^P<0.01 vs. control, ^##^P<0.01 vs. TNFα alone, n=6-8.


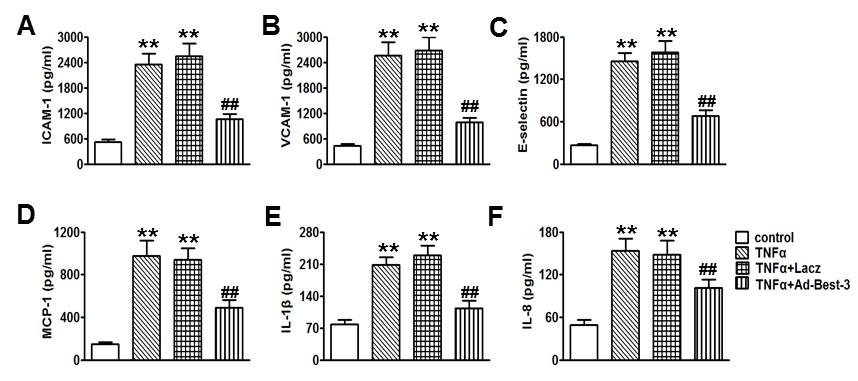
**Figure S6**

**Figure S6. Best-3 reduced TNFα-induced secretion of inflammatory mediators *in vivo*. (A-F)** the levels of ICAM-1 (A), VCAM-1 (B), E-selectin (C), MCP-1 (D), IL-1β (E) and IL-8 (F) in serum harvested form mice after treatment mentioned in method section were determined by ELISA assay. ^**^P<0.01 vs. control, ^##^P<0.01 vs. TNFα alone, n=10.
